# Involvement of rRNA biosynthesis in the regulation of *CUC1* gene expression and pre-meristematic cell mound formation during shoot regeneration

**DOI:** 10.3389/fpls.2014.00159

**Published:** 2014-04-28

**Authors:** Naoki Shinohara, Iwai Ohbayashi, Munetaka Sugiyama

**Affiliations:** Botanical Gardens, Graduate School of Science, The University of TokyoTokyo, Japan

**Keywords:** shoot regeneration, rRNA biosynthesis, WD40 repeat protein, CUC1, RID2, RID3

## Abstract

At an early stage of shoot regeneration from calli of Arabidopsis, pre-meristematic cell mounds develop in association with localized strong expression of *CUP-SHAPED COTYLEDON* (*CUC*) genes. Previous characterization of *root initiation-defective 3* (*rid3*), an Arabidopsis mutant originally isolated as being temperature-sensitive for adventitious root formation, with respect to shoot regeneration implicated *RID3* in the negative regulation of *CUC1* expression and the restriction of cell division in pre-meristematic cell mounds. Positional cloning has identified *RID3* as a WD40 repeat protein gene whose molecular function was not investigated before. Here we performed *in silico* analysis of RID3 and found that RID3 is orthologous to IPI3, which mediates pre-rRNA processing in *Saccharomyces cerevisiae*. In the *rid3* mutant, rRNA precursors accumulated to a very high level in a temperature-dependent manner. This result indicates that RID3 is required for pre-rRNA processing as is IPI3. We compared *rid3* with *rid2*, a temperature-sensitive mutant that is mutated in a putative RNA methyltransferase gene and is impaired in pre-rRNA processing, for seedling morphology, shoot regeneration, and *CUC1* expression. The *rid2* and *rid3* seedlings shared various developmental alterations, such as a pointed-leaf phenotype, which is often observed in ribosome-related mutants. In tissue culture for the induction of shoot regeneration, both *rid2* and *rid3* mutations perturbed cell-mound formation and elevated *CUC1* expression. Together, our findings suggest that rRNA biosynthesis may be involved in the regulation of *CUC1* gene expression and pre-meristematic cell-mound formation during shoot regeneration.

## Introduction

In the course of shoot regeneration from calli, callus-derived cells are newly assembled into shoot apical meristems (SAMs), which eventually develop into adventitious buds. This SAM construction can be regarded as a *de novo* cell organization process free from the regulation by pre-existing structures in the plant body, and exemplifies the spontaneous nature of plant organogenesis.

In the model plant *Arabidopsis thaliana* (Arabidopsis), vigorous shoot regeneration can be induced from either root or hypocotyl segments by a two-step tissue culture procedure consisting of pre-culture on callus-inducing medium (CIM), which is rich in both auxin and cytokinin, and the subsequent culture on shoot-inducing medium (SIM), which also contains both auxin and cytokinin but in a higher cytokinin/auxin ratio (Valvekens et al., 1988; Akama et al., 1992). The CIM culture triggers cell proliferation in the stele of explants and leads to callus formation. Transfer onto SIM changes cell proliferation pattern in the calli so that spots of active proliferation emerge and give rise to cell mounds on the callus surface. Those cell mounds further form SAMs. These morphological changes are associated with expression of genes that regulate SAM establishment and maintenance, such as *CUP-SHAPED COTYLEDON 1* (*CUC1*), *CUC2*, *WUSCHEL* (*WUS*), *CLAVATA 3* (*CLV3*), and *SHOOT MERISTEMLESS* (*STM*) (Cary et al., 2002; Gordon et al., 2007). Local, strong expression of *CUC1* and *CUC2* precedes expression of the other SAM regulator genes in calli cultured on SIM, and marks pre-meristematic cell mounds. Considering that *CUC*s are indispensable in *STM* expression and SAM establishment during embryogenesis (Aida et al., 1999), this expression pattern suggests that spatially controlled *CUC* expression may be the key event in an early phase of shoot regeneration.

We previously isolated a series of temperature-sensitive mutants of Arabidopsis with adventitious root formation as an index phenotype (Konishi and Sugiyama, 2003). Of these mutants, *root initiation defective 3* (*rid3*) and *root growth defective 3* (*rgd3*) have been characterized mainly with respect to shoot regeneration in tissue culture (Tamaki et al., 2009). In *rgd3* explants, high temperature suppresses expression of *CUC1* and *STM* and inhibits cell-mound formation severely. Contrastingly, in *rid3* explants, high temperature elevates expression of *CUC1* and *STM*, expands their expression regions, and leads to the formation of irregularly large mounds. *RGD3* encodes BATF1, a specific kind of TATA-binding protein-associated factor, and *RID3* encodes a WD40 repeat protein (Tamaki et al., 2009). Both *RGD3* and *RID3* are expressed uniformly in calli but they become different in expression patterns after calli are transferred onto SIM. In fact, *RGD3* is mainly expressed in developing cell mounds whereas *RID3* is expressed outside the cell mounds. These findings implicate *RGD3* and *RID3* in the positive and negative regulation of *CUC1* expression and pre-meristematic cell-mound formation, respectively.

Our collection of temperature-sensitive mutants contains *rid2*, an rRNA biosynthesis-defective mutant (Ohbayashi et al., 2011). This mutant accumulates large amounts of pre-rRNA processing intermediates due to a mutation in a putative RNA methyltransferase gene. In the previous phenotypic analysis in tissue culture, effects of the *rid2* mutation were examined with a focus on callus formation, but not for shoot regeneration (Ohbayashi et al., 2011).

Here, to approach molecular mechanisms underlying pattern formation at an early stage of shoot regeneration, we investigated the function of *RID3*. Phylogenic analysis of *RID3* led to the notion that *RID3* might be involved in pre-rRNA processing, which was confirmed by the measurement of rRNA precursors in *rid3*. We compared *rid3* with the other pre-rRNA processing-defective mutant *rid2* for seedling development and shoot regeneration-related phenotypes. On the basis of these results, we discuss a developmental role of rRNA biosynthesis during shoot regeneration.

## Materials and methods

### *In silico* analysis of RID3

For reciprocal best-hit cluster analysis, eukaryotic protein sequences related to Arabidopsis RID3 were retrieved from six well-annotated genome datasets, and two prokaryotic sequences similar to Arabidopsis RID3 were retrieved from the non-redundant dataset of NCBI (http://www.ncbi.nlm.nih.gov). Sequences most similar to those RID3-related sequences in each genome were also retrieved. Thus, total 16 sequences were collected from 8 genome datasets. All the 256 pairs were then examined whether they were in the reciprocal best-hit relationship, in which the best-hit sequence in another genome matches its query sequence as the best-hit in its own genome. For phylogenetic tree construction, protein sequences in an RID3 reciprocal best-hit cluster along with reference sequences, which include the most similar RID3 homolog in the Arabidopsis genome and its best-hits in some other genomes, were analyzed by the maximum-likelihood method implemented in the Phylogeny.fr platform (Dereeper et al., 2008). WD-40 repeat domains of RID3 were identified by the Pfam program (http://pfam.sanger.ac.uk/) as significant Pfam-A matches.

### Plant materials

The Landsberg *erecta* (L*er*) strain of Arabidopsis was used as the wild type in this study. *rid2* (*rid2-1*), *rid3*, and *rgd3* (*rigd3-1*) were all derived from mutagenized L*er* plants (Konishi and Sugiyama, 2003). *CUC1p::CUC1:GUS*, a β-glucuronidase (GUS) reporter gene for monitoring *CUC1* expression, was described previously (Takada et al., 2001). L*er* and *rid2* plants carrying *CUC1p::CUC1:GUS* were used for expression analysis.

Plants were cultured aseptically under continuous illumination (70–90 μmol/m^2^/s) on MS medium (Murashige and Skoog, 1962) that was supplemented with 1% (w/v) sucrose, buffered to pH 5.7 with 0.05% (w/v) 2-(morpholin-4-yl)ethanesulfonic acid (MES), and solidified with 0.8% (w/v) gellan gum, unless otherwise indicated.

### Tissue culture

Tissue-culture experiments were performed at various temperatures under continuous illumination (15–25 μmol/m^2^/s) as described previously (Tamaki et al., 2009) with slight modifications. Donor plants for tissue culture were grown at 19°C for 12 days under continuous dim light (approximately 10 μmol/m^2^/s) on MS medium supplemented with 1% (w/v) sucrose, buffered to pH 5.7 with 0.05% (w/v) MES, and solidified with 1.5% (w/v) agar. Hypocotyl segments were excised from these plants and pre-cultured on CIM at 19°C for 6 days. Then explants were transferred onto SIM for the induction of shoot regeneration and cultured at 19, 22, 25, or 28°C. CIM and SIM were modified from B5 medium (Gamborg et al., 1968). They were supplemented with 2% (w/v) glucose, buffered to pH 5.7 with 0.05% (w/v) MES, and solidified with 0.25% (w/v) gellan gum. CIM contained 0.5 mg/l (2,4-dichlorophenoxy)acetic acid and 0.1 mg/l kinetin as phytohormones, whereas SIM contained and 0.04 mg/l indole-3-acetic acid and 1.5 mg/l *N*^6^-Δ^2^-isopentenyladenine.

### RNA gel blot analysis

Total RNA was prepared from the wild-type and mutant plants grown at various temperatures by using TRIzol reagent (Invitrogen) and separated electrophoretically on agarose gel. The electrophoresed RNAs in a gel were blotted onto a positively charged nylon membrane (Roche Diagnosis), and the membrane was hybridized with a digoxigenin-labeled probe specific for ITS1 of pre-rRNA (Supplementary Table S1). Hybridization signals were detected with the DIG system (Roche Diagnosis) according to the manufacturer's protocol.

### Real-time PCR analysis

Total RNA was prepared in three biological replicates from the wild-type and mutant explants cultured at various temperatures by using TRIzol reagent (Invitrogen), and reverse-transcribed using PrimeScript RT reagent kit with gDNA Eraser (TaKaRa) according to the manufacturer's protocol. The obtained cDNA was used as templates for real-time PCR. Real-time PCR was performed with gene-specific primers (Supplementary Table S1) using SYBR Premix ExTaq II (TaKaRa) on StepOne Real Time PCR System (Life Technologies). The thermal cycling program was an initial denaturation at 95°C for 30 s followed by 40 cycles of amplification reactions, each consisting of 5-s denaturation at 95°C and 30-s annealing/extention at 60°C. The expression level of a target gene was normalized to that of *TUBULIN ALPHA-4 CHAIN* (*TUA4*).

### Histochemical detection of GUS activity

The color development of the GUS activity in plant specimens was performed as described by Ohtani and Sugiyama (2005). The color-developed samples were cleared with an 8:1:2 w/v/v mixture of chloral hydrate, glycerin and water before observing with a microscope equipped with Nomarski optics (BX50-DIC; Olympus).

## Results

### Identification of RID3 orthologs

**Figure 1:**
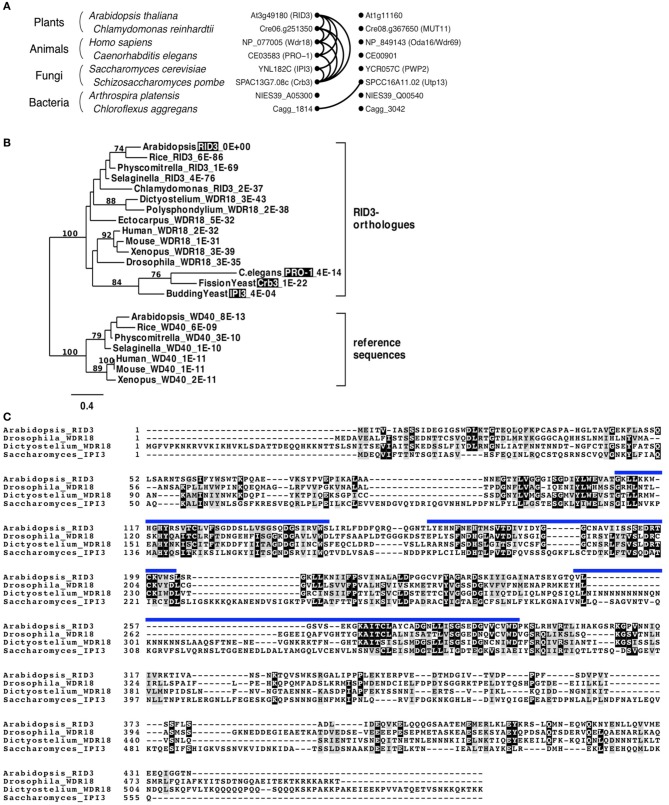
**Identification of RID3 orthologs. (A)** Graphical representation of a reciprocal best-hit cluster. Dots and curve lines represent protein sequences and reciprocal best-hit relationships, respectively. Each row contains two similar sequences from one species. **(B)** A maximum-likelihood tree generated by protein sequences. Numbers on branches indicate bootstrap values when more than 70 in 100 repetitions. For each taxon, an organism name, a protein name alias (shaded if its mutant has been reported), and an *E*-value to RID3 are shown (for details of each sequence, see Supplementary Table S2). **(C)** Clustal W alignment of amino acid sequences of Arabidopsis RID3, Drosophila WDR18, Dictyostelium WDR18, and budding yeast (Saccharomyces) IPI3. Identical and similar residues are shaded with black and gray, respectively. Blue lines represent WD-40 repeat domains of RID3.

Pairwise comparisons of genes for searching best hits in genome datasets of different organisms can identify reciprocal best-hits and their clusters. Genes in such clusters are believed to be, at least in many cases, in orthologous relationships (Kristensen et al., 2011). Therefore, we explored a *RID3*-containing cluster of reciprocal best-hits by performing multiple pairwise comparisons between well-annotated genome databases of various organisms, and found that a *RID3*-containing cluster lay within the eukaryote domain (Figure 1A). We then used sequences in such a cluster for phylogenetic tree construction. As reference sequences of the phylogenic tree, we chose the Arabidopsis homolog most similar to RID3 and its best-hit sequences in some other genomes, in consideration of the usefulness of duplicated gene pairs to specify the root position in a phylogenic tree (Iwabe et al., 1989). As shown in Figure 1B, the sequences of a *RID3*-containing cluster formed one distinct clade, within which the topology largely agreed with the generally accepted evolutionary history of the eukaryote lineage, although the clade grouping the *Caenorhabditis elegans* and yeast sequences together seems to be influenced by “long-branch attraction,” a common artifact in sequence-based phylogenetic tree construction (reviewed by Bergsten, 2005). Nonetheless, the well-supported branch (100%) of the sequences in the cluster indicates that the member sequences, including RID3, IPI3 of budding yeast *Saccharomyces cerevisiae* (Saccharomyces), and Pro-1 of *C. elegans*, were orthologous to each other. This conclusion is consistent with the previous large-scale analysis for orthologous group identification (Tatusov et al., 2003; KOG0646). In addition, we found that the Arabidopsis closest homolog lay outside the clade of RID3 orthologs despite the much higher BLAST-based similarity (*E*-value: 8E-13) than that (4E-04) of IPI3 to RID3 (Figure 1B).

Figure 1C shows alignment of amino acid sequences among RID3 of Arabidopsis, WDR18s of *Drosophila melanogaster* (Drosophila) and *Dictyostelium discoideum* (Dictyostelium), and IPI3 of Saccharomyces. Although the similarity between IPI3 and the other three proteins is relatively low, there are many conserved amino-acid residues within the WD40 repeat region and also in its flanking regions.

### Impairment of pre-rRNA processing in *rid3*

IPI3 of budding yeast has been best studied at the molecular level among the RID3 orthologs, and was demonstrated to participate in rRNA biosynthesis as a component of a pre-rRNA processing factor complex (Krogan et al., 2004). The orthologous relationship between RID3 and IPI3 therefore suggests that RID3 may be involved in rRNA biosynthesis. To test this possibility, we measured rRNA precursors in wild-type and *rid3* plants grown at various temperatures by RNA gel blot analysis. For comparison, *rid2*, a temperature-sensitive mutant that is mutated in a putative RNA methyltransferase gene and impaired in pre-rRNA processing (Ohbayashi et al., 2011), and *rgd3*, a temperature-sensitive mutant whose mutation lies in the BATF1 gene and is thus supposed to be irrelevant to rRNA biosynthesis (Tamaki et al., 2009), were also used.

**Figure 2:**
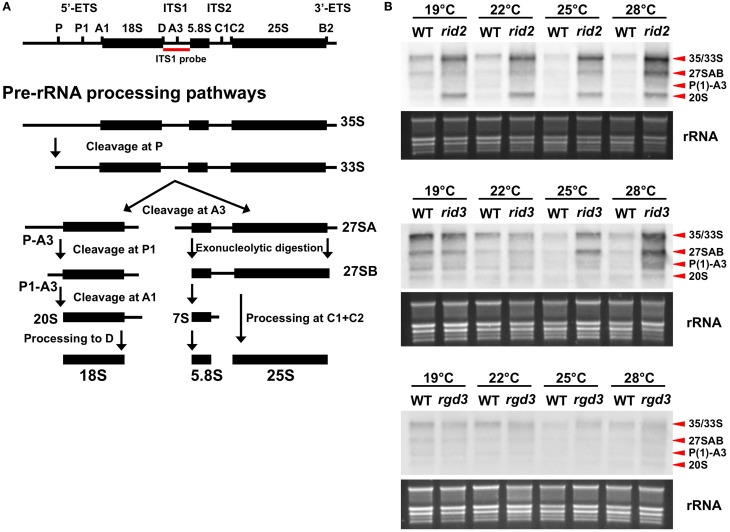
**Effect of *rid3* mutation on rRNA precursor accumulation**. **(A)** Pre-rRNA processing pathway in Arabidopsis based on Zakrzewska-Placzek et al. (2010). **(B)** RNA gel blot analysis of rRNA precursors. Seedlings of *rid3* and wild type (WT) were cultured at 19, 22, 25, or 28°C for 16 days after sowing. Seedlings of *rid2* and *rgd3* were also cultured under the same condition as references. Total RNA samples were prepared from these seedlings and 3 μg/lane of RNA was electrophoresed. RNA gel blot analysis was performed with a probe specific to ITS1. rRNA bands visualized by staining with GelRed (Biotium) are shown as an equal loading control.

As the result, rRNA precursors of several different sizes such as 27S and 33S/35S accumulated to a very high level compared to the wild-type level in the *rid2* and *rid3* plants grown under high temperature conditions but not in the *rgd3* plants (Figure 2). The accumulation of rRNA precursors in *rid3* was strongly influenced by the growth temperature and the unusually high-level accumulation occurred at higher temperatures than in *rid2*, which accumulated large amounts of rRNA precursors even at 19°C. These findings indicate that RID3 was required for proper processing of rRNA precursors as are IPI3 and RID2. It is noteworthy that in *rid3* rRNA precursor levels relative to the wild-type levels were correlated well with its growth temperatures. In the wild type, rRNA precursor levels were slightly higher at lower temperatures, suggesting that changes in ambient temperature can affect rRNA biosynthesis.

Accumulation patterns of rRNA precursors were somewhat different between *rid2* and *rid3*. *rid3* accumulated P(1)-A3 pre-rRNA more abundantly than 20S pre-rRNA, whereas *rid2* accumulated 20S pre-rRNA more abundantly than P(1)-A3 pre-rRNA (Figure 2B). This result showed that pre-rRNA processing events were differentially affected by the *rid2* and *rid3* mutations, probably reflecting different molecular functions of RID2 and RID3.

### Comparison of *rid3* with *rid2* for their effects on seedling shoot development and shoot regeneration

**Figure 3:**
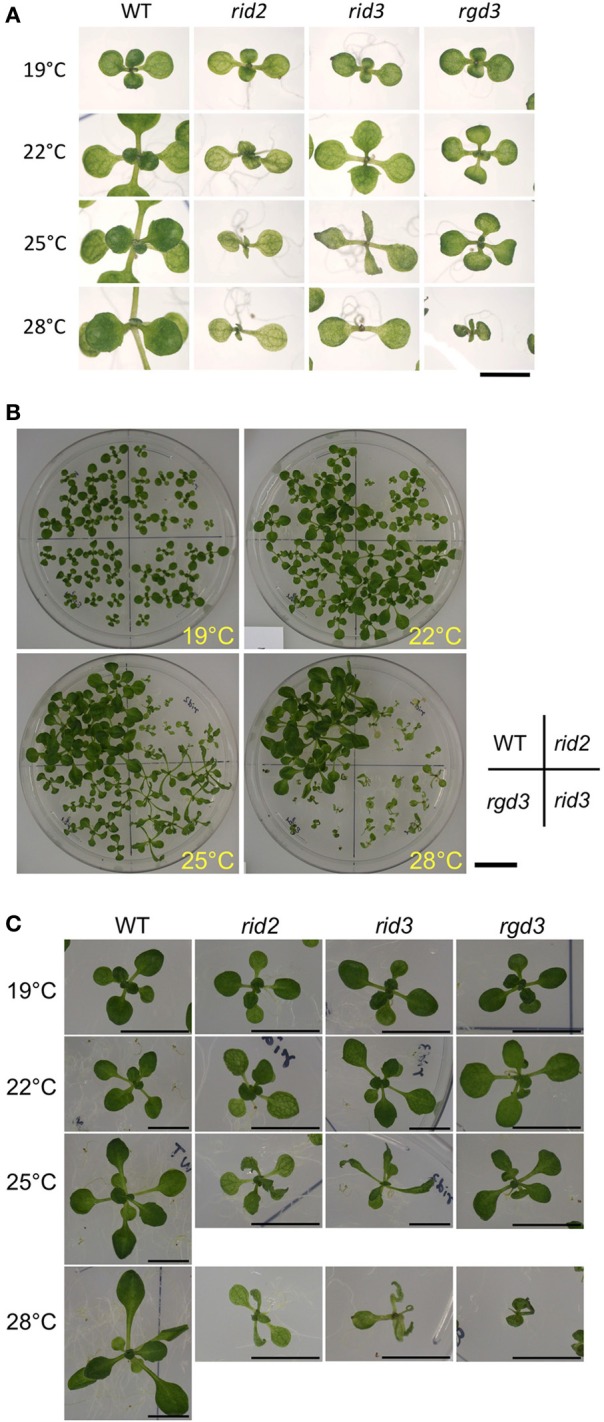
**Morphological phenotypes of seedlings cultured at various temperatures. (A)** Cotyledons and first several true leaves of 12-day-old seedlings of *rid2*, *rid3*, *rgd3*, and wild type (WT) grown at 19, 22, 25, or 28°C. Bar = 5 mm. **(B)** Overall appearances of 16-day-old plants. Bar = 2 cm. **(C)** Leaf phenotypes 16-day-old plants. Bars = 1 cm.

To examine the relationship between the altered activity of pre-rRNA processing and morphological phenotypes of *rid3*, we compared *rid3* with wild type, *rid2*, and *rgd3* for seedling development at various temperatures. When seedlings were grown at 19°C, neither of *rid2*, *rid3* nor *rgd3* seedlings were much different in appearance from those of wild type (Figure 3). Contrastingly, at 28°C, all of those mutants grew much slower than wild type and displayed severe abnormalities. At intermediate temperatures, *rid2* and *rid3* showed seemingly common phenotypes (Figure 3C). In fact, at 25°C, both *rid2* and *rid3* developed twisted, narrower, and more pointed leaves with deeper indentations compared with wild type. Those leaves were paler, which made venation more obvious. Those morphological phenotypes in leaves were not found in *rgd3*. Although the aforementioned phenotypes of *rid2* and *rid3* were seemingly the same, those mutants considerably contrasted in their leaf length when grown at 25°C. At that temperature, *rid2* leaves were much shorter than those of wild type, whereas *rid3* leaves developed longer than those of wild type.

**Figure 4:**
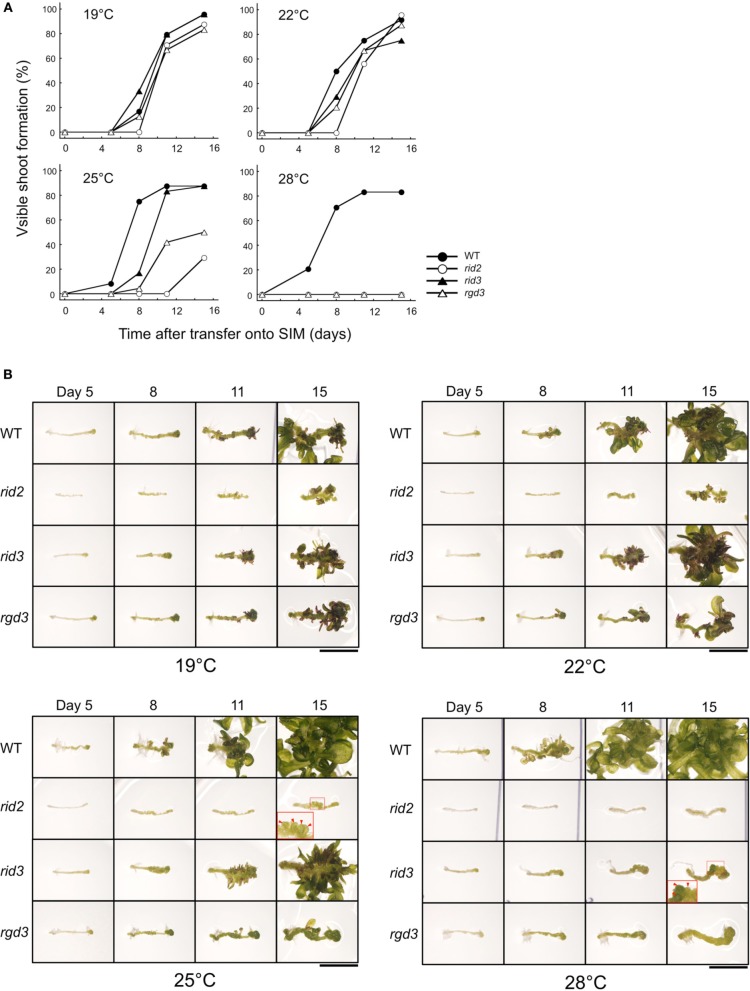
**Shoot regeneration-related phenotypes of hypocotyl explants cultured at various temperatures. (A)** Time course of shoot regeneration from hypocotyl explants of *rid2*, *rid3*, *rgd3*, and wild type (WT). Explants were cultured on SIM at 19, 22, 25, or 28°C after 6 days of pre-culture on CIM at 19°C. *n* = 24. **(B)** Morphological changes of explants during culture on SIM. Photographs in each row indicate the same explant at different times. Insets show magnified images of the squared regions. Red arrowheads indicate irregularly large mounds of cells. Bar = 5 mm.

For comparing shoot regeneration between *rid2* and *rid3*, we pre-cultured excised hypocotyl explants of those mutants along with wild type and *rgd3* on CIM at 19°C, and then cultured them on SIM at various temperatures. When cultured at 25°C or lower temperatures, *rid3* explants regenerated shoots at high frequency (Figure 4A). At 28°C, however, they were unable to regenerate shoots and formed irregularly large mounds of cells instead of adventitious buds (Figure 4B), as reported before (Tamaki et al., 2009). As *rid3* mutation did, *rid2* mutation inflicted temperature-dependent defect on shoot regeneration, but its influence was severer than that of *rid3* mutation (Figure 4). Even at 25°C, *rid2* explants scarcely regenerated adventitious shoots and formed irregular mounds of cells, (Figure 4B). In *rid2* explants cultured at 28°C, basic cell proliferation appeared to be suppressed. Unlike *rid3* and *rid2*, *rgd3* explants cultured on SIM do not form irregularly large mounds of cells (Tamaki et al., 2009; Figure 4B). In this respect, *rid3* was similar to *rid2*, but different from *rgd3*.

In the culture condition employed in this experiment, adventitious root formation took place frequently besides shoot regeneration. This root formation was not strongly inhibited by *rid3* and *rgd3* mutations even at 28°C, whereas *rid2* mutation reduced the root formation in a temperature-dependent manner (Supplementary Table S3).

### Effects of the *rid2* and *rid3* mutations on CUC1 and STM expression during SIM culture

**Figure 5:**
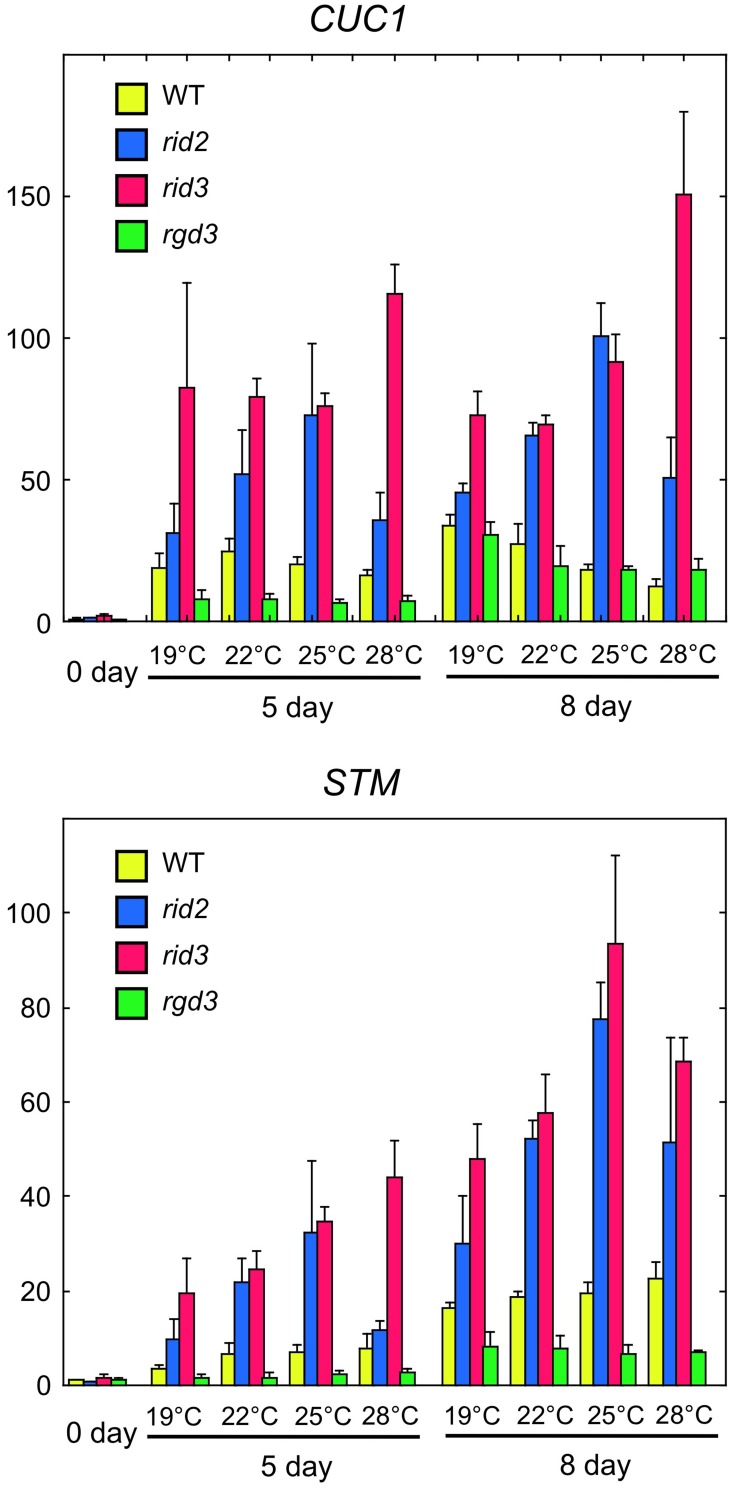
**Real-time PCR analysis of expression of *CUC1* and *STM* in hypocotyl explants cultured on SIM**. Hypocotyl explants of *rid2*, *rid3*, *rgd3*, and wild type (WT) were cultured on SIM at 19, 22, 25, or 28°C after 6 days of pre-culture on CIM at 19°C. Total RNA was prepared from explants at 0, 5, and 8 day after transfer onto SIM and used for real-time PCR analysis of *CUC1* and *STM* expressions. Means and standard deviations of normalized relative quantities (relative to the mean of the 0-day samples of wild type) of biological triplicates are shown.

Expression of *CUC1* and *STM* genes in cultured explants is closely associated with shoot regeneration (Cary et al., 2002; Gordon et al., 2007). To compare *rid2* and *rid3* mutations for their effects on the expression of *CUC1* and *STM*, we performed real-time PCR analysis using cDNA samples prepared from *rid2*, *rid3*, *rgd3*, and wild-type explants that were cultured on SIM at various temperatures after pre-culture on CIM at 19°C. In the wild-type explants, culture on SIM induced strong expression of *CUC1* and later *STM* at any temperature (Figure 5). *rid3* mutation elevated those expression levels, particularly at 28°C. *rid2* mutation also elevated *CUC1* and *STM* expressions, with the maximum effect occurring at 25°C. Unlike *rid2* and *rid3* mutations, *rgd3* mutation did not elevate but suppress *CUC1* and *STM* expressions. Because *CUC1* activates *STM* expression (Hibara et al., 2003), elevated expression of *STM* in *rid2* and *rid3* may be attributed, at least partly, to the elevated expression of *CUC1* in those mutants.

**Figure 6:**
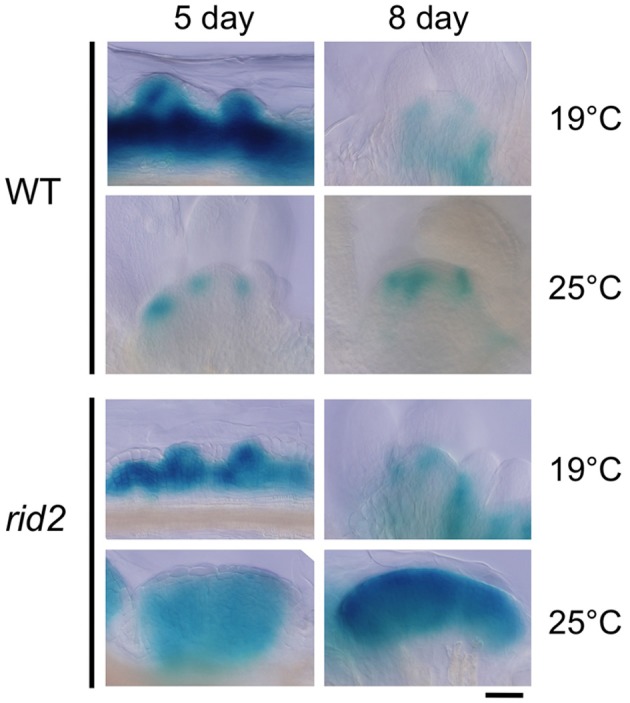
***CUC1p::CUC1:GUS* expression in hypocotyl explants of *rid2* and wild-type cultured on SIM**. Hypocotyl explants of *rid2* and wild-type (WT) carrying *CUC1p::CUC1:GUS* were cultured on SIM at 19°C or 25°C after 6 days of pre-culture on CIM at 19°C and subjected to histochemical detection of GUS activity. Bar = 50 μm.

Spatial patterns of *CUC1* expression during shoot regeneration were examined by the GUS reporter gene *CUC1p::CUC1:GUS* introduced into wild-type and *rid2* backgrounds (Figure 6). Explants cultured on SIM at 19°C or 25°C for 5 or 8 days before GUS-signal detection were observed by light microscopy. In wild-type background explants cultured at 19°C for 5 days on SIM, strong GUS signals were detected in a broad area inside the calli and the signals locally extended to some spots of the callus surface. Those spots were mostly overlapped with mounds of relatively small cells. After 5 days culture at 25°C, wild-type background explants were forming SAMs, where GUS signal was strong at boundaries between the SAM and initiating leaf primordia and declined at the center. In *rid2* background explants cultured at 19°C, two different patterns of GUS signal were observed: one was signal localized similarly to the pattern found in wild-type background explants, and the other was diffused or expanded signal pattern. When cultured at 25°C for 5 days, *rid2* background explants showed ill-localized or diffused/expanded pattern of GUS signal, and after prolonged culture, they formed large mounds of GUS-positive cells. These results suggested that *rid2* mutation may alter spatial control of *CUC1* expression to lead to irregular development of cell mounds.

## Discussion

In this study, we identified RID3 orthologs from diverse taxa of eukaryotes by in-depth analysis of genome databases. Each eukaryotic genome, as far as we investigated, contained only one RID3 ortholog, and WD40 proteins whose similarities to the RID3 ortholog were as low as that of RID3 to the closest Arabidopsis homolog (Supplementary Table S2). It is therefore suggested that the common ancestral gene of *RID3* and its orthologs has had a molecular function distinct from other WD40 repeat protein genes before the divergence of eukaryotes, and each RID3 ortholog has stayed as a single gene in a genome since then. IPI3, the RID3 ortholog of budding yeast is a component of a pre-rRNA processing complex, and the *IPI3* shutoff experiment has revealed its function in pre-rRNA processing (Krogan et al., 2004). PRO-1, the RID3 ortholog of *C. elegans*, has also been reported to mediate pre-rRNA processing (Voutev et al., 2006). On the basis of temperature-dependent accumulation of rRNA precursors in *rid3*, we showed that RID3 was involved in pre-rRNA processing. Therefore the common function of the RID3 orthologs may be pre-rRNA processing in eukaryotes.

*rid2* is a temperature-sensitive mutant isolated along with *rid3* in the same screening (Konishi and Sugiyama, 2003), and has defects in pre-rRNA processing (Ohbayashi et al., 2011). Comparison of *rid2* and *rid3* revealed that these mutants shared many phenotypes that were not found in *rgd3*, a temperature-sensitive mutant unrelated to rRNA biosynthesis. Both *rid2* and *rid3* seedlings formed narrow, pointed, and twisted leaves with pale green in color, conspicuous indentations, and obvious venations at intermediately high temperatures. At least some of these leaf phenotypes (such as a pointed and narrow shape and conspicuous indentations) are often found in ribosome protein mutants and ribosome biogenesis mutants (e.g., Van Lijsebettens et al., 1994; Byrne, 2009; Horiguchi et al., 2011). Degenhardt and Bonham-Smith (2008) demonstrated, by knock down experiments of RPL23aA, a large-subunit ribosomal protein, that the ribosomal protein transcript level is correlated with the developmental alterations including leaf phenotypes. Therefore the leaf phenotypes of *rid2* and *rid3* seem to result mostly from a partial shortage or dysfunction of the ribosome. The common morphological features observed in the leaves of ribosome-related mutants also suggested that some particular processes of leaf development might be vulnerable to minor shortage or dysfunction of the ribosome.

Our previous characterization of *rid3* focusing on shoot regeneration and analysis of expression patterns of *RID3* and *CUC1* have implied that RID3 might have a role of negatively regulating *CUC1* expression and cell proliferation to achieve proper development of pre-meristematic cell mounds (Tamaki et al., 2009). The present study revealed that RID3 participated in rRNA biosynthesis and might illuminate a functional link between rRNA biosynthesis and *CUC1* expression and pre-meristematic cell mound formation at an early stage of shoot regeneration. In fact, both *rid2* and *rid3* mutations increased the expression level of *CUC1* and induced irregularly large cell mound formation. Additionally, GUS reporter analysis revealed that, relative to the wild type, *rid2* explants displayed a more diffused or expanded pattern of *CUC1* expression, which was accompanied by large cell mound formation. This effect of *rid2* mutation on *CUC1* expression pattern was similar to the effect of *rid3* mutation (Tamaki et al., 2009). These findings collectively suggest that the level of rRNA biosynthesis limited by processing factors such as RID2 and RID3 may be involved in the restriction of *CUC1* expression and cell proliferation required for development of pre-meristematic cell mounds into SAMs.

Szakonyl and Byrne (2010) studied in detail *rpl27ac-1D*, a semi-dominant mutation in the ribosomal protein gene *RPL27aC* of Arabidopsis, and reported that this mutation perturbs apical domain patterning during embryogenesis. Of interest, ectopic and expanded expression of *CUC2* and *STM* coincided with the morphological defect of embryogenesis in the *rpl27ac-1D* mutant (Szakonyl and Byrne, 2010). This and our findings together raise the possibility that the ribosomal biogenesis including rRNA synthesis played an important role in broad aspects of shoot apical pattern formation through the regulation of the *CUC*-*STM* pathway.

It remains a big question how the ribosome, fundamental machinery essential for translation, regulates expression of specific genes and specific processes of plant development. In animals, perturbations of ribosome biogenesis are considered to contribute to “nucleolar stress” and trigger a stress response pathway mediated by interactions between ribosomal proteins, the oncoprotein MDM2, and the tumor-suppressor protein p53 (reviewed by Deisenroth and Zhang, 2010). If plants have a similar pathway, it might be a good candidate for a mechanistic link between ribosome biogenesis and selective regulation of gene expression and developmental processes. Another possibility is that a mechanism independent of such stress-sensing pathway might be responsible for the regulatory functions of ribosomes. For example, mRNA sequence features such as the length of 5′-untranslated regions and the presence of upstream ORFs affect ribosome loading efficiency (Kawaguchi and Balley-Serres, 2005) and thus might cause differences in the sensitivity of translation to the amount of ribosomes. Future studies on the significance of ribosome biogenesis, its surveillance system, and differential regulation of translation in the context of plant development would provide insights into hidden aspects of molecular mechanisms of *de novo* pattern formation.

### Conflict of interest statement

The authors declare that the research was conducted in the absence of any commercial or financial relationships that could be construed as a potential conflict of interest.

## Supplementary material

Supplementary Table S1 **List of primers used for probe synthesis and real-time PCR**.

Supplementary Table S2 **Sequences used for phylogenetic tree construction**.

Supplementary Table S3 **Percentages of root-forming explants after 8 days of culture on SIM**.
